# Dog ownership satisfaction determinants in the owner-dog relationship and the dog's behaviour

**DOI:** 10.1371/journal.pone.0204592

**Published:** 2018-09-20

**Authors:** Ineke R. van Herwijnen, Joanne A. M. van der Borg, Marc Naguib, Bonne Beerda

**Affiliations:** Department of Animal Sciences, Behavioural Ecology Group, Wageningen University and Research, Wageningen, The Netherlands; Universidade do Porto Instituto de Biologia Molecular e Celular, PORTUGAL

## Abstract

Dog ownership satisfaction relates to the quality of life of both owner and dog, and when seriously compromised may even lead to dog abandonment. Knowledge on determinants of dog ownership satisfaction is limited, obstructing solutions for promoting satisfaction, and here we quantified causes making dog owners less than very satisfied with their dog. We focused on the owner perceived relationship with the dog, unwanted dog behaviour, and dog obedience class attendance. The study population included only few seriously dissatisfied dog owners, preventing discrimination of multiple levels below ‘very satisfied’. Consequently, existing relationships in the entire population may have been missed or underestimated and the findings apply specifically to dog owners that are relatively contented with dog ownership. Nine hundred seventy-seven Dutch dog owners completed an online questionnaire and we found the probability of being very satisfied to associate with all three subscales of the Monash Dog Owner Relationship Scale. Most strongly with perceived costs of ownership and less so with shared activities between owner and dog, and perceived emotional closeness to the dog. Aggression and/or disobedience related directly to high perceived ownership costs and to an increased probability of being less than very satisfied. Interaction effects indicated that dog disobedience was less influential on ownership satisfaction at high levels of aggression. Surprisingly, dog ownership satisfaction was unrelated to dog obedience class attendance, raising questions about the effectiveness of these classes in establishing satisfying dog-owner relationships. Training aids used during classes could play a role here, as choke chain use associated with high perceived costs and increased probabilities of being less then very satisfied with dog ownership. Ownership satisfaction in relatively contented dog owners, seems more influenced by unwanted dog behaviour and perceived costs of ownership, than by perceived emotional closeness to the dog, shared activities and dog obedience class attendance.

**Highlights**

- Dog ownership satisfaction associated with the Monash Dog Owner Relationship Scale.
- Perceived dog ownership costs associated with ownership satisfaction most strongly; and with a dog’s aggression and disobedience.
- Dog obedience class attendance did not associate with dog ownership satisfaction.

## Introduction

Dog ownership has the potential to support personal development and well-being by means of the dog fulfilling its owner’s psychological needs for autonomy, competence, and relatedness [1]. Dogs, ‘on the other side of the leash’, benefit too, for instance from enjoying interactions with their owner and humans in general. Dogs actively sought human proximity when interacted with by petting [2] and the presence of a human caretaker lowered stress in dogs facing novel environments [3]. However, there is variation in the nature of owner-dog relationships. For instance, dogs can be seen as loving companions or merely as toys or status enhancers, which influences the extent to which both parties benefit from the relationship [4]. Dog ownership satisfaction reflects several aspects of the owner-dog relationship such as owner-dog attachment strength [5]. When this attachment strength is compromised it increases the risk of the dog being relinquished [6]. Yet it remains unclear what makes dog owners (very) satisfied with their dog and here we quantify the relative importance of obvious determinants of such satisfaction, based on known risk factors for dog abandonment such as unwanted dog behaviour, the owner perceived relationship with the dog and attendance to dog obedience classes [5, 7, 8, 9]. Abandonment as an extreme consequence of ownership dissatisfaction constitutes a serious issue. In the US, millions of animals enter shelters each year and some are even presented there to be euthanized [10]. For the Netherlands, numbers of more than 12,000 dogs entering shelters were projected, on an estimated population of 1.8 million dogs [11]. Reasons for abandoning a dog and being dissatisfied with owning it may be diverse, including an imbalance between how a dog is expected to behave and actually does [7]. Unwanted behaviour, meaning behaviour that is undesired by the dog owner and/or hazardous to others (e.g. biting), is thought to contribute strongly to dog abandonment [12, 13]. Biting people and being perceived as overly active, increased a dog’s risk of abandonment in a comparative study with 2,092 people who relinquished their dog to a shelter and 3,434 people who kept their dog [14].

Unwanted behaviour in companion animals is common, at least in the eyes of companion animal owners. Forty-three percent of Dutch companion animal owners reported at least one behaviour problem in their dog or other companion animal [15] and for dogs this percentage may be higher, for example given the 68% of Italian trainee guide dog puppy walkers who reported undesirable behaviour [16]. Aggressive behaviour directed at unfamiliar people was the main complaint in 140 dog owners seeking advice from a veterinary hospital behaviour service (48%), followed by aggressive behaviour directed at familiar people (43%) and at other dogs (40%) [17]. The high proportion of cases of dog aggression presented to behavioural clinics underlines how such behaviour is problematic to dog owners. Disobedience is another behaviour in dogs that owners consider problematic, and main behavioural issues reported by 203 Australian dog owners were overexcitement (63%) and jumping up on people (56%) [18]. Excessive aggression or disobedience in dogs troubles owners and makes them look for solutions. It is less clear though, what the quantitative impact on ownership satisfaction is of more moderate aggression and/or disobedience in common privately-owned dogs that are not specifically studied for behaving problematically.

Dog ownership satisfaction may be influenced differently in dogs surrendered at shelters and presented at behavioural clinics than in dogs that only moderately express risk factors such as aggression or disobedience. Studying common dogs on owner-dog relationship dimensions, is therefore valuable. Such owner-dog relationship dimensions are often assessed with the Monash Dog Owner Relationship Scales (MDORS) [19, 20, 21]. This tool consists of 28 questions measuring on three subscales [22]. The subscales cover perceived emotional closeness to the dog, perceived costs of owning the dog in terms of effort and financial costs and shared activities between owner and dog [22]. More information on these and other determinants of dog ownership satisfaction in relatively satisfied dog owners, facilitates the identification and use of early warning signals for a compromised owner-dog relationship. Also, dog obedience class attendance is generally thought to improve this relationship, but scientific findings are inconsistent about the effect of such classes on for instance achieved levels of desired dog behaviour [23]. Much can be learned about what determines dog ownership satisfaction and which factors prevent the long-term dissatisfaction that obstructs owners and dogs to benefit from their relationship. Knowing what makes owners especially satisfied with owning a dog can be a stepping stone towards strategies in support of an optimal owner-dog relationship.

Here we studied factors that make dog owners less than ‘very satisfied’ with their dog, focussing on the owner to dog relationship and the dog’s behaviour. Our main goal was to identify determinants of ownership satisfaction by means of quantifying associations between ownership satisfaction, owner-dog relationship dimensions (MDORS), unwanted dog behaviour, dog obedience class attendance and use of training aids.

## Methods

### Questionnaire

Reports by dog owners were used to evaluate candidate determinants of dog ownership satisfaction and to quantify strengths of existing relationships. We collected data via an online questionnaire and participants were recruited via the internet, including websites frequently visited by dog owners, internet fora on dog topics and social media channels such as Facebook. Also, flyers about the online questionnaire were handed out at shelters, veterinary clinics and by dog professionals (dog trainers and dog behavioural therapists). Anyone owning a dog was eligible for the research and we did not practice criteria for inclusion or exclusion.

The online questionnaire introduction explained the purpose of the research and the study did not involve treatments or interventions in the life of respondents or their dogs. The questionnaire was not repeated, meaning it did not interfere significantly with normal daily life and did not include questions that were psychologically stressful. This exempts the study from review by our ethics committee, according to the guidelines of Wageningen University Medical Ethics Review Committee (Medisch Ethische Toetsingscommissie van Wageningen University, METC-WU). Informed consent was not obtained as respondents chose to participate freely via internet and the purpose of the research was stated at the start of the online survey.

Details on the participating dog owners (*N =* 977) and their dogs are presented in the results section. The questionnaire was in Dutch, but see S1 Appendix for the English translation, and consisted of 54 miscellaneous questions on topics such as dog characteristics, way of acquisition, the dog’s behaviour and living conditions. Respondents were asked to fill out the survey with one particular dog in mind. Quantitative questions were typically answered on a five-point Likert scale, like a dog’s tendency to aggress or disobey. Aggression was assessed by eight questions, on dog behaviour in daily life situations that involved (un)familiar people and dogs, including possessiveness and territoriality. Aggressive behaviour scores were expressed as a percentage of the theoretical maximum, given the number of questions that the participant filled out. For the assessment of dog obedience we followed a similar procedure using the two questions ‘Indicate how often your dog comes immediately when called’ and ‘Indicate how often your dog is overly active by jumping up/pushing against you’. Dog obedience class attendance was measured with the five answer categories ‘not at all’, ‘<8 weeks’, ‘2–6 months’, ‘6–12 months’ and ‘>12 months’, but for further analyses the scores were expressed on a binary scale with 1 representing the four levels of class attendance and 0 indicating no attendance. There were 28 Monash Dog Owner Relationship Scales (MDORS) questions on the owner-dog relationship, which were taken from Dwyer et al. [22] and used to assess the owner perceived emotional closeness to the dog (MDORS^Close^), the owner perceived costs of owning the dog (in terms of effort and finance), time and efforts in general (MDORS^Cost^), and the engagement in shared activities (MDORS^Shared^). MDORS scores were calculated for each of the three subscales by combining item scores into a percentage of the theoretical maximum. The MDORS^Cost^ subscale was expressed reversely, with high scores reflecting low perceived costs and a strong owner-dog relationship. Dog ownership satisfaction was assessed by asking ‘How satisfied are you with your dog?’, with the answer categories being ‘not at all satisfied’ (1), ‘not very satisfied’ (2), ‘moderately satisfied’ (3), ‘satisfied’ (4) and ‘very satisfied’ (5). Satisfaction scores were skewed towards high levels of satisfaction and answers were therefore expressed as a binary number with 1 being ‘very satisfied’ and 0 being ‘less than very satisfied’. A total of 977 surveys was analysed, but questions could be left unanswered and sample size varied across questions as indicated in the results section. The MDORS questions were presented as an optional extra and sample size was lowest for tests that involved these items (down to *N =* 889).

### Statistical analyses

We used logistic regressions with dog ownership satisfaction as the binary response variate (y), using GenStat (18^th^ edition) software. We tested the associations between dog ownership satisfaction and the owner-dog relationship in a logistic regression model with the three MDORS subscales as explanatory variables with the inclusion of two-way interactions. Interactions that were not significant were omitted from the statistical model. We ran separate logistic regression models on the relation between ownership satisfaction and the scores for a dog’s aggressive behaviour and/or disobedience as explanatory variables, again including two-way interaction. Means (± SE) predicted by the logistic regressions are presented for the range of the 50% middle values (the two central quartiles) of the explanatory variables (MDORS^Close^, MDORS^Cost^, MDORS^Shared^, aggression, disobedience). This means that effect sizes in the dependent variables (response variates) were illustrated for the independent variables’ range of common values. Also, we tested if the owners’ use, or not, of training aids such as food, play, clicker or correction chain explained dog ownership satisfaction. Logistic regressions on ownership satisfaction were done per training aid. Finally, we tested if the response variate dog obedience class attendance, expressed on a binary scale with having attended classes as 1 and never having attended a class as 0, associated with the earlier described explanatory variables MDORS subscales, aggressive behaviour and disobedience, tested in a logistic regression model with main effects only.

An approach, similarly as described above, was used for further analyses of owner perceived relationships, as expressed in the MDORS scores. We ran ANOVAs with the subscale scores for MDORS^Close^, MDORS^Cost^ and MDORS^Shared^ as dependent variables to test for effects of the independent variables dog aggression and disobedience (two-way ANOVA, including interaction), obedience class attendance (one-way), and training aid use (one-way). Aggression and disobedience were expressed as percentages and the other independent variables were expressed as factors with two levels (yes, no).

To facilitate interpretations based on the logistic regressions and ANOVAs we tested for associations between explanatory variables with Pearson’s and Spearman’s rank correlations, including the three MDORS subscales, and the dogs’ aggressive behaviour and disobedience. Only Spearman’s test outcomes are presented as Pearson’s tests gave similar results.

## Results

### Characteristics of participating dog owners and their dogs

The study sample of 977 dog owners consisted mostly of experienced dog owners (74%, *N =* 715), owning more than one dog (59%, *N* = 572), with 26% being first time owners (*N =* 257). We found no statistically significant difference for dog ownership satisfaction level between first time owners and experienced owners. Participants reported on dogs of various breeds with 51% of the dogs being females (209 intact, 272 neutered) and 49% males (274 intact, 192 neutered). The majority of dogs were reported to have a normal energy level, with an average(±SD) 2.1±0.9 on a five-point scale of very calm (score 0) to highly energetic (4). Three quarters of participants walked their dog for more than an hour (75%, *N =* 734), on a typical weekday, played more than ten minutes with their dog (76%, *N =* 740) and left the dog alone for no more than four hours (78%, *N =* 759).

### Dog ownership satisfaction and perceived relationship dimensions

Participants (*N =* 977) were typically satisfied with their dog, and the average (±SD) dog ownership satisfaction score was 4.7±0.7 on a scale of 1 (lowest: *N =* 9; via 2: *N =* 5, 3: *N =* 34, 4: *N =* 191) to 5 (highest, *N =* 738), and 0.8±0.4 when expressed on a binary scale, with very satisfied as 1 (*N =* 738) and less than very satisfied as 0 (*N =* 239). The owner-dog relationship was rated similarly for shared activities and emotional closeness, with average MDORS scores of 68% of the theoretical maximum and rated relatively high for low perceived costs (87%, see Table 1 for details). Associations between MDORS subscale scores were all significant, but explained less than 8% of the variation, with Spearman’s rank correlations of *r*_s_ = 0.25, P<0.001, *N =* 889 for MDORS^Close versus Cost^, *r*_s_ = 0.27, P<0.001, *N =* 889 for MDORS^Close versus Shared^, *r*_s_ = 0.13, P<0.001, *N =* 889 for MDORS^Cost versus Shared^.

**Table 1:** Monash Dog Owner Relationship Scale (MDORS) scores. Average owner perceived relationship scores (MDORS, *N =* 889) in a study population of Dutch dog owners derived from an online questionnaire, for the overall sample and split between the highest level of dog ownership satisfaction and less satisfied owners.

Monash Dog Owner Relationship Scale (MDORS)	For all respondents - average%±SD (range)	For respondents scoring 1 (very satisfied) - average%±SD (range)	For respondents scoring 0 (less than very satisfied) - average%±SD (range)
**MDORS^Shared^**	68.4±11.6%	69.2±11.4%	65.8±12.0%
	(17–100%)	(17–100%)	(28–92%)
**MDORS^Close^**	68.2±15.5%	69.6±15.6%	64.0±14.5%
	(10–100%)	(10–100%)	(18–100%)
**MDORS^Cost^**	87.2±10.8%	89.1±9.5%	81.4±12.6%
	(42–100%)	(44–100%)	(42–100%)

**Fig 1:**
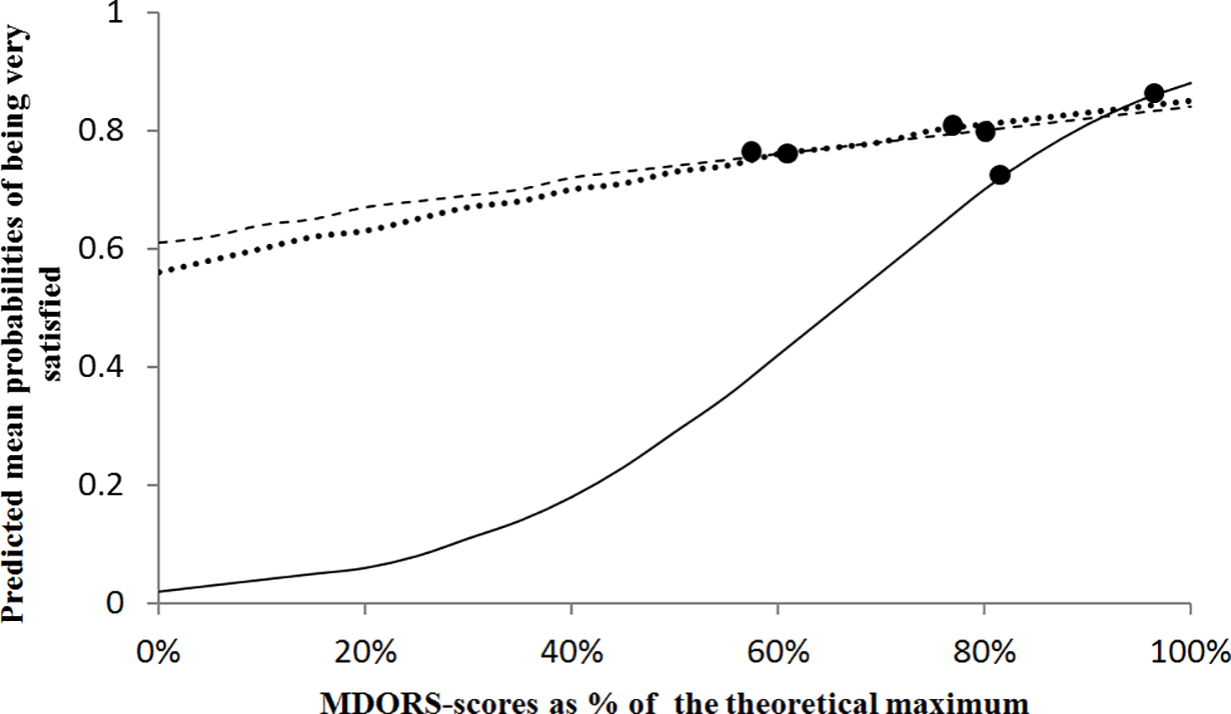
Probabilities of being very satisfied with owning a dog in relation to MDORS scores. Predicted mean probabilities of being very satisfied with owning a dog (y-axis) in 889 Dutch dog owners in relation to their self-reported emotional closeness (dashed line, P = 0.04), perceived costs of ownership (solid line, P<0.001) and number of shared activities with the dog (dotted line, P = 0.04). The MDORS scores are expressed as percentages of the theoretical maximum and associated significantly with dog ownership satisfaction. The marked points indicate the range of 50% middle scores for each of the MDORS subscales.

The probability of being very satisfied with owning one’s dog was significantly associated with all three MDORS subscales in a logistic regression with 889 records (Fig 1). Predicted mean probabilities of being very satisfied increased significantly (logistic regression, P = 0.04) from 0.76±0.02 to 0.80±0.02 with MDORS^Close^ scores increasing from 58 to 80%, that is across the range of 50% middle values (the two central quartiles). Similarly, increasing MDORS^Shared^ scores from 61 to 78% raised probabilities on being very satisfied from 0.76±0.02 to 0.80±0.02 (logistic regression, P = 0.04). The strongest effect was noted for MDORS^Cost^ and across the range of 81 to 97% the probabilities of being very satisfied increased from 0.71±0.02 to 0.86±0.02 (logistic regression, P<0.001). So, within our sample of mostly satisfied dog owners, dog ownership satisfaction related directly to a good owner to dog relationship and especially to low perceived costs of having the dog.

### Aggression and disobedience

Increasing levels of aggressive behaviour significantly lowered the chance of owners being very satisfied with their dog. Increases of this behaviour over the range of middle values (the two central quartiles), that is from 3 to 19% of the theoretical maximum, decreased predicted mean probabilities of being very satisfied from 0.84±0.01 to 0.71±0.02 (on a scale from 0 to 1; logistic regression, P<0.001, *N =* 976). The average(±SD) aggression score in the study sample was 12.1±12.6% (range 0–78.1%, *N =* 977; S2 Appendix provides details on prevalence of aggression and obedience in the sample).

Obedience scores too, associated significantly with ownership satisfaction with probabilities of being very satisfied increasing from 0.70±0.02 to 0.84±0.01 (on a scale from 0 to 1) with obedience scores increasing from 63 to 88% (logistic regression, P<0.001, *N =* 972). The average(±SD) obedience score in the study sample was 74.4±17.8% (range 0–100%, *N =* 977).

**Fig 2:**
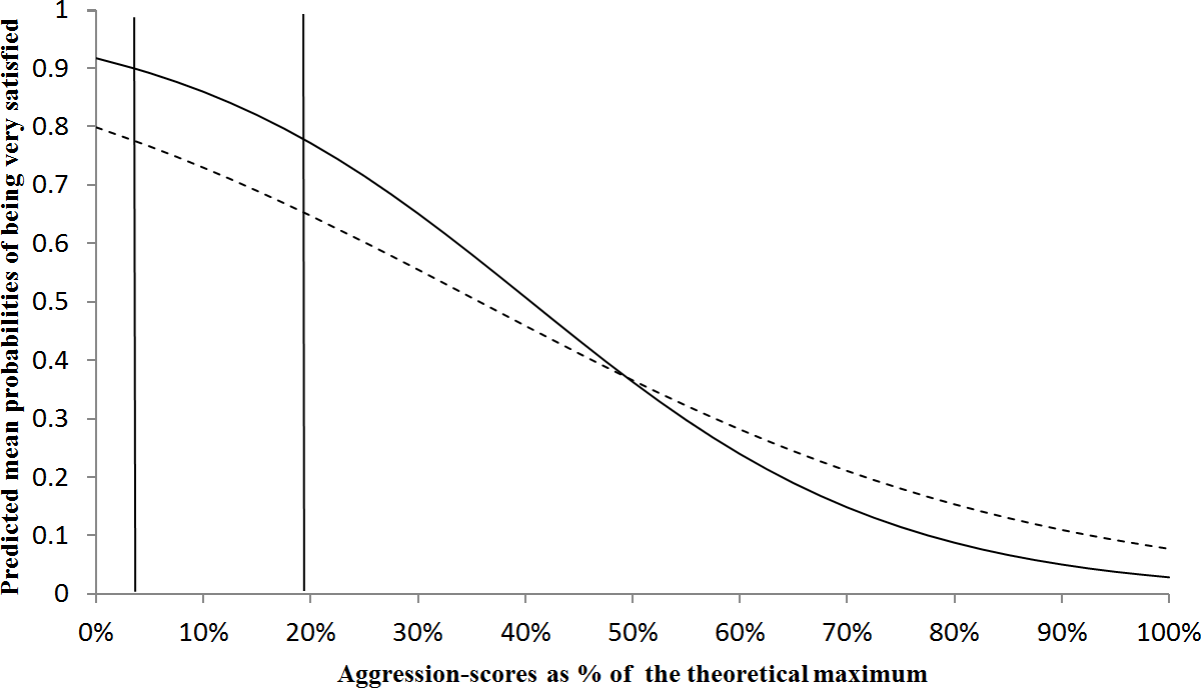
Probabilities of being very satisfied with owning a dog in relation to the dogs’ aggression and disobedience. Predicted mean probabilities of being very satisfied with owning a dog (y-axis) in 974 Dutch dog owners in relation to the dogs’ aggression and disobedience in daily life (two-way interaction P<0.05). Behaviour scores are expressed as percentages of the theoretical maximum with aggressions on the x-axis and the two lines representing high obedience (88%, solid line) and low obedience (63%, dashed line), with the two vertical lines indicating the range of 50% middle scores for aggression (3–19%).

Aggressive behaviour and disobedience combined, could be expected to have particularly strong effects on dog ownership satisfaction, which was confirmed by a significant two-way interaction (logistic regression, P = 0.005, *N =* 974). Fig 2 shows that in relatively disobedient dogs, the inverse relationship between the dogs’ aggressive behaviour and owners’ satisfaction was linear, whereas it was mirror S-shaped in relatively obedient dogs. In the 50% range of middle values (the two central quartiles) for aggressive behaviour (3 to 19%), dog disobedience lowered ownership satisfaction, but the strength of this association waned with increasing levels of aggression. In dogs showing more serious aggression, with scores over 20%, the influence of dog obedience was predicted to be less relevant to ownership satisfaction. Predictions for extreme cases of aggressive behaviour are speculative as these were rare in the present study population.

Aggressive behaviour and disobedience were tested for associations with MDORS scores as dependent variables in two-way ANOVAs. Neither aggression, disobedience, nor interactions between these, related significantly to MDORS^Close^ or MDORS^Shared^ (P>0.1). Aggression did relate (inversely) to MDORS^Cost^ and over the range of common values for aggression (3–19%) the MDORS^Cost^ percentages decreased with 2% from 88.2±0.4% to 86.4±0.4% (F_(1,890)_ = 14.0, P<0.001). Similarly, obedience scores over the range of 63–88% associated with a 3% increase in MDORS^Cost^ from 85.8±0.4% to 88.8±0.4% (F_(1,890)_ = 33.6, P<0.001; P = 0.3 for the two-way interaction between aggression and obedience). MDORS^Cost^ was scaled reversely and high owner perceived costs of dog ownership thus coincided with high levels of dog aggression and disobedience.

To test for entanglement of explanatory variables we performed Spearman rank correlations between aggression and disobedience, and between these two variables and the three MDORS subscales. Outcomes explained less than 5% of variance and were significant only for the dog’s aggressive behaviour and disobedience (*r*_s_ = 0.14, P<0.001, *N =* 889), and MDORS^Cost^ and aggressive behaviour (*r*_s_ = -0.15, P<0.001, *N =* 889) or disobedience (*r*_s_ = -0.21, P<0.001, *N =* 889). The limited strengths of associations between these explanatory variables do not raise major concerns about entanglements determining the interpretation of statistical outcomes. They seem to reflect mainly how the dog’s aggressiveness and disobedience lead to higher owner perceived cost.

### Dog obedience class attendance and use of training aids

Most participants had at some time attended dog obedience classes with their dog (78%, *N =* 757 out of 971), resulting in an average(±SD) 0.78±0.42 on a binary scale. We tested with logistic regression if dog obedience class attendance (yes versus no) was associated with dog ownership satisfaction, but results were not significant (P = 0.3).

Class attendance did not explain variation in perceived costs of dog ownership (MDORS^Cost^ ANOVA P = 0.6), but did associate with lower emotional closeness and more shared activities. MDORS^Close^ percentages were a predicted mean 67.6±0.6% for dog owners who had attended obedience classes compared to 70.3±1.1% for those who had not (F_(1,890)_ = 4.7, P = 0.03). MDORS^Shared^ percentages were a predicted mean 69.1±0.4% for owners who had attended classes compared to 65.8±0.8% for those who had not (F_(1,884)_ = 11.5, P = 0.001).

Regarding the use of training aids, most dog owners trained their dogs using play (57%, *N =* 559), food (75%, *N =* 734) or play and food (89%, *N =* 874). A clicker was used by 28% (*N =* 276) of the dog owners and a correction chain by 8% (*N =* 83). The use of a correction chain (yes versus no) was significantly associated with a reduced probability of being very satisfied with the dog (logistic regression, P = 0.005, *N =* 976), with predicted mean probabilities dropping from 0.77±0.01 for owners not using correction chains to 0.63±0.05 for those who did. The significant relationship between the use of a correction chain and perceived costs of dog ownership, was in line with this finding. MDORS^Cost^ predicted means (reversed scale) were 84.0±1.3% for dog owners using a correction chain and 87.5±0.4% for those who did not (ANOVA F_(1,892) =_ 7.6, P = 0.006; P>0.1 for MDORS^Shared^ and MDORS^Close^).

No significant associations with dog ownership satisfaction were found for play, food or clicker use. The outcomes of logistic regressions are questionable if there are only few occurrences for (some) combinations of factors, with 10 being a frequently reported minimum. The lowest count here was at least 31, as found for the combination of being less than very satisfied and making use of a correction chain, with all other counts ≥52.

The use of play, food or clicker as training aid did not significantly explain variation in MDORS^Cost^ scores (ANOVA P>0.3), whereas the use of food associated with low emotional closeness and high levels of shared activities. MDORS^Close^ predicted means were 67.6±0.6% for dog owners who used food as a training aid and 70.1±1.0% for those who did not (F_(1,894)_ = 4.3, P = 0.038). MDORS^Share^ predicted means were 68.8±0.5 in owners using food and 67.0±0.8% in those who did not (F_(1,888)_ = 3.9, P = 0.049). Finally, MDORS^Close^ predicted means were 69.0±0.6% for dog owners who used a clicker as training aid and 66.4±1.0% for those who did not (F_(1,894)_ = 5.3, P = 0.022; P>0.07 for MDORS^Share^).

## Discussion

Knowledge of what determines dog ownership satisfaction may be utilized for strategies to improve the owner-dog relationship, possibly even lowering abandonment rates of dogs [5, 6]. Here we quantified the effects of several candidate satisfaction determinants from 977 Dutch dog-owner reports. In our typical study sample of mostly satisfied dog owners, the probability of being very satisfied with one’s dog was in part explained by the perceived relationship with the dog, in particular with the perceived costs of owning it, and with the dog’s aggressive behaviour and disobedience. The latter two factors interacted, with high levels of aggression overshadowing the effects of disobedience on ownership satisfaction. Aggression and disobedience, as main effects, associated with high perceived costs only, in line with the strong relationship between perceived costs and dog-ownership satisfaction. Unexpectedly we did not find dog ownership satisfaction to associate with dog obedience class attendance. Our findings come from a study population of highly satisfied dog owners and do not necessary apply to the more serious levels of dissatisfaction. Most likely this typical sample has affected the quantifications of effects, with a bias towards underestimates, and reduced the power to detect relationships due to the underrepresentation of the severe cases of dissatisfaction. An argument for considering the significant findings applicable to the entire population of Dutch dog owners is the correspondence of outcomes with known reasons for owners to abandon their dog. Nevertheless, the present results should be viewed in the specific context of dog owners who were relatively contended with owning their dog.

We applied a binary divide of dog ownership satisfaction levels, discriminating between being very satisfied and less than that. The reason for this was the high percentage of dog owners (76%) reporting the highest level of satisfaction. The actual situation will be less positive as we assume our study sample of volunteers recruited by mainly (social) media to be skewed towards people with positive opinions about having a dog and thus willing to make the effort of filling out a research questionnaire on dogs. Unintentionally, much research on dog ownership is done with highly engaged dog owners [21, 24], because participating in research requires effort, which average or less engaged dog owners are less likely to invest. Presently, science has not found an easy solution to this issue. We expect our research to present a relatively rosy picture of dog ownership satisfaction mainly due to this selection bias. The effect of long known questionnaire response artefacts, such as impression management, acquiescence bias and/or midpoint-responding [25, 26] likely had a minor effect on our findings. We assume our research topic of factors influencing dog ownership satisfaction, not sensitive to the degree known to influence questionnaire research [27]. We searched for demographics of the Dutch dog owner population, but we were unable to find statistics that could be used for comparison with our study group. Two hundred and thirty-nine dog owners were less than very satisfied with owning their dog, compared to 738 who were, and such an imbalance potentially causes low counts for combinations of factors in the logistic models. As a rule of thumb 10 outcome events per predictor variable are considered as a minimum, though this is subject to debate with suggestions that the rule can be relaxed [28], actually does not prevent unreliable estimates [29] or at least requires further validation [30]. The minimal number of events in our study was 31 with other counts over 51, giving us little concern about the reliability of the logistic model estimates. The present findings apply to dog owners with relatively high levels of engagement with their dog and dog ownership, but it may be questioned if findings extrapolate to the remainder of the Dutch dog owner population. The associations tested by us were fitted (curvi-)linearly, as there were no strong reasons for deviating from this basic approach. It may be argued that the found effects on the probability of being very satisfied with one’s dog may turn out to be different from those on the probability of being very dissatisfied. This seems unlikely though, as the explanatory variables predicting dog ownership satisfaction were selected by us for being known risk factors of dog abandonment. Apparently, a dog’s (slight) tendency to aggress or disobey, as well as a dog owner’s perception of the costs of owning a dog, determine ownership satisfaction in satisfied owners as well. In this, dog unwanted behaviour and perceived costs of ownership relate directly, with associations existing for both aggression and disobedience. Such association with unwanted behaviour were absent for emotional closeness and amount of shared activities. Consequently, unwanted behaviour and high perceived costs of ownership have potential as early warning signals of a less optimal relationship that could, in the end, result in dog abandonment. Furthermore, these factors can identify points of action for improving the owner-dog relationship before issues have become serious and the owner very dissatisfied.

Dog obedience classes seem an obvious way to prevent misbehaviours in dogs and to build a strong owner-dog relationship. Such classes are designed to increase dog obedience levels and were seen to lower aggressive behaviour when classes were followed with young dogs [31]. Also, dog obedience class attendance reduced the odds of an adopted dog being returned to the organisation adopting it out [7] and class attendance shortly after acquiring a dog increased the chance of continuing dog ownership [9]. Surprisingly, we found no relation between dog obedience class attendance and dog ownership satisfaction or perceived costs. Class attendance did associate with more shared owner-dog activities, which may in part reflect the shared activity of attending the classes themselves. The unexpected inverse relationship between class attendance and emotional closeness to the dog was weak (P = 0.03) and requires further underpinning. Variation in obedience class content and quality likely leads to varying dog outcomes and owner-dog relationship effects. One such variation may involve training aids that are advertised during obedience classes. We found the use of a correction chain to associate with higher perceived costs of ownership and lower dog ownership satisfaction. Weak associations (P≈0.04) were found for the use of food in training, coinciding with more shared activities and lower levels of reported emotional closeness to the dog. Such findings raise questions about how obedience classes teach owners to influence dog behaviour and with what outcomes for the owner-dog relationship. Australian dog owners were surveyed on obedience class experiences and although the 178 owners reported that the classes resulted in better training skills, they did not necessarily provide desired dog behavioural outcomes, for instance with regard to aggression [23]. Improving dog training skills in dog owners is important, but it may be that having a well-behaved, non-aggressive, dog and being knowledgeable on or skilled in the use of appropriate training aids, is of greater importance to dog ownership satisfaction.

The highest level of dog ownership satisfaction in our study associated logically with all three measured aspects of the owner-dog relationship, as assessed with the MDORS [22], but we found an important quantitative variation. Perceived costs changed the probability of being very satisfied with 15% where this was only 4% for shared activities and emotional closeness, across the range of 50% middle values (the two central quartiles). The same factor of perceived costs has been associated with oxytocin levels in ten male Labrador Retrievers [19]. The dogs were owned by middle-aged females and perceived costs associated in the expected direction with blood oxytocin levels (*r* = -0.8), which were in turn related to the owner’s oxytocin levels [19]. Oxytocin is a neuropeptide that facilitates attachment and bonding in several animal species [32, 33] and stimulates social behaviour [34]. Low perceived costs of having a dog thus seem a strong indicator of a good owner-dog relationship, reflecting in oxytocin levels and ownership satisfaction with the dog.

Dog aggression and disobedience associated inversely with ownership satisfaction to a similar degree, with respective decreases in probability of being very satisfied of 13 and 14%, across the range of 50% middle values. A companion animal’s behaviour is of major importance to ownership satisfaction. In small animals such as rabbits, mustelids and rodents, unwanted behaviours were associated with lowered ownership satisfaction, notwithstanding high overall mean satisfaction levels of 8.6 out of 10 [35]. In dogs, good behaviour may be particularly important, as North-American adopters (*N =* 343) of both dogs and cats reported how good behaviour of their animal associated with ownership satisfaction, with associations being stronger for dogs than for cats [13]. Unwanted behaviour is a main reason for relinquishment [6, 9, 12] and near 60% of dogs were returned for reasons of misbehaviour in a large scale six-month follow-up study of 4,500 rehomed dogs (15% were returned) [7]. Some unwanted behaviours may be more disturbing to common dog owners than others. Seventy-five percent of 74 Australian adopters of shelter dogs wished their dog to show less unwanted behaviour, such as that related to fear, but 57% reported to overall be very satisfied with their dog’s behaviour [36]. Aggression is particularly troublesome behaviour. An inverse relation between aggressive behaviour in dogs and ownership satisfaction was found in a study on 645 Australian dog owners, with owners of friendly (and obedient) dogs being especially satisfied [37]. Problematic behaviours in dogs may interact when affecting ownership satisfaction. Here we found (dis)obedience to be of little effect when aggression levels were high, nearing scores of 50% of the theoretical maximum. Such high aggression levels are rare. Within the range of common levels the effects of disobedience and aggression were near independent and additive, meaning we found no indications that comorbidity potentiated the impact of single misbehaviours.

Clearly other factors than the ones discussed so far determine dog ownership satisfaction, including the personalities of both owner and dog. Interpersonal relationship satisfaction is reduced by a person’s neuroticism, where agreeableness and extraversion increase satisfaction by means of substantiating empathy [38, 39, 40]. Similarity of personality associates with higher relationship satisfaction, more so than does complementarity [41], though inconsistent findings exist [39]. In the owner-dog relationship similarity of personality may also be advantageous. Dog ownership satisfaction was directly related to owner perceived complementarity for warmth of their dogs and themselves [42]. The 449 dog owners reported on dog ownership satisfaction, emotional attachment and their dog adding positivity to life. Warmth was considered one of two main components of social behaviour with complementary dominance having no influence on dog ownership satisfaction [42]. Also, complementarity of owner and dog in willingness to ‘share, loving to run outside, acting destructive and getting along with others’, explained dog ownership satisfaction in a study on 88 dog owners [43].

Prospective dog owners will have expectations of ownership, which may or may not match reality. Candidate adopters of dogs and cats reported that the behaviour of their future companion animal is of prior concern to them [44]. Expectations of the role pets should play vary with gender, with having children and with ownership experience, as demonstrated in study population 343 adopters of shelter dogs and cats [45] with similar findings in a more recent survey of 877 Australians on their ‘ideal dog’ [46]. Common expectations of a dog’s phenotypical traits are about it being medium sized and short haired, having good health and behaving socially and obedient [46]. One unrealistic expectation of dog ownership may regard the amount of effort it takes to care for it. An unexpectedly high care effort is a major reason for returning adopted dogs [7] and this matches with the presently found relatively strong relationship between perceived costs and ownership satisfaction.

Unwanted behaviour in the dog was found to coincide with high perceived costs and such behaviour may worsen a suboptimal situation. With an owner already perceiving costs of the relationship as high, the additional effort required to counter unwanted behaviour in the dog, may be insurmountable. The resulting risk of relinquishment makes it of prime concern to prevent and solve such dog behavioural issues at its early stages. The role for dog obedience classes in reaching this objective, may be less straightforward than expected, and it may be questioned if obedience classes today reach their full potential to promote wanted behaviour in dogs and contribute to a satisfying owner-dog relationship.

## Supporting information

S1 Appendix Questionnaire items, other than MDORS. Questionnaire items other than Monash Dog Owner Relationship Scale (MDORS), gathering information on the dog and dog ownership.
(PDF)

S2 Appendix Prevalence of dog aggression and obedience in 968 to 972 Dutch dog owners. Prevalence of dog aggression and two obedience behaviours based on the owner’s assessments of different situations (*N =* 968 to 972, with precise sample sizes between brackets).
(PDF)
