# Supplementary material for: Dog ownership satisfaction determinants in the owner-dog relationship and the dog's behaviour
Source: PLoS One. 2018 Sep 20;13(9):e0204592. doi: 10.1371/journal.pone.0204592 (PMC6147508; doi:10.1371/journal.pone.0204592)
Supplement: S1 Appendix — Questionnaire items other than Monash Dog Owner Relationship Scale (MDORS), gathering information on the dog and dog ownership. (PDF) [file pone.0204592.s001.pdf]

S1 Appendix - Questionnaire items, other than MDORS

Questionnaire items other than Monash Dog Owner Relationship Scale (MDORS), gathering information on the dog and dog ownership

General questions on the dog:

1. Of which breed or type is your dog?

[open]

2. Does your dog have an FCI-pedigree?

[yes/no]

3. Which age is your dog?

[<4 months/4-6 months/6-12 months/1-2 years/2-5years/5-10 years/>10 years]

4. Is your dog female or male?

[female/male]

5. Is your dog intact?

[yes/no]

6. Which coat type does your dog have?

[short, thick coat/short, thin coat/middle length, thick coat/middle length, thin coat/long thick coat/long thin coat]

Questions on dog ownership:

7. How satisfied are you with your dog?

[not at all satisfied/not very satisfied/moderately satisfied/satisfied/very satisfied]

8. Does your dog meet the wishes you had when you bought it?

[yes/no]

9. Which reason for having a dog fits your situation best?

[for company/because it is pretty/for the children/as companion on walks/to practice a dog sport/for guarding purposes/for work, other than guarding/other]

10. How would you type your dog for:

    a. energy level

[highly active/active/normal/calm/very calm]

    b. stubbornness versus mellowness

[very stubborn/stubborn/normal/mellow/very mellow]

    c. stability versus sensitivity?

[very stable/stable/normal/sensitive/very sensitive]

11. How did you prepare for becoming a dog owner?

[not at all/information from relatives/information from breeder/information from internet/reading books/information from organization/other

12. How much time was in between the moment of deciding to have a dog and actually getting it?

[<1 week/1-4 weeks/1-6 months/>6 months]

13. Is this your first dog (being a child younger than 16 years not included)?

[yes/no]

14. Did you grow up as a child younger than 16 years with a dog?

[yes/no]

15. Through which channel did you acquire your dog?

[single nest ‘in house’ breeder/breeding kennel or shed/store or market/home delivered/shelter/relatives/foreign dog placement organization/Dutch rehoming organization/own breeding/self-obtained from abroad]

16. Did you choose this way of acquiring the dog purposely?

[yes/no]

17. Did you visit the place/organisation where you bought your dog more than once?

[yes/no]

18. Did the seller have a conversation with you of more than 30 minutes upon buying the dog?

[yes/no]

19. Did the seller contact you after acquisition to learn how things are with the dog?

[yes/no]

20. Did you use after care, if provided by the seller?

[yes/no]

21. Did your dog reside at a foster home, before your acquisition of it?

[yes/no]

22. To your knowledge, was your dog ever in a shelter?

[yes/no]

23. What was the age of the dog at acquisition?

[<7 weeks/7-12 weeks/3-6 months/6-12 months/1-2 years/2-5years/5-10 years/>10 years]

24. Was your dog checked for disease or disorder before you acquired it?

[yes/no]

25. Have you ever contemplated rehoming your dog with a relative or via a shelter?

[yes/no]

26. How much did you pay for your dog at acquisition?

[<100 euro/100-200 euro/200-500 euro/500-1000 euro/>1000 euro]

27. If you had another chance of acquiring a dog, would you buy one?

[yes/no]

28. Would it be of a same or different breed or type than you have now?

[yes/no]

29. Is your dog presently suffering from disease or disorder?

[yes/no]

30. Has your dog previously suffered from disease or disorder?

[yes/no]

  a. If so, which?

  [open]

31. Indicate how often your dog:

  a. Exercises off leash in populated area
  b. Exercises off leash outside out-side populated area
  c. Comes immediately when called

[never/nearly never/sometimes/often/always]

32. How many dogs live with you, next to the dog you are filling out this survey for?

[0/1/2/>2]

33. Indicate how often your dog:

  a. Pulls on leash
  b. Pees or poops in the house
  c. Runs away
  d. Runs after game or stock
  e. Runs after bikers/runners/riders
  f. Barks/wines/howls when alone
  g. Destructs
  h. Is overly active by jumping up/pushing against you
  i. Is hyperactive or restless
  j. Begs for food or attention
  k. Steals food
  l. Eats poop from other animals
  m. Rolls in poop, dead animals or garbage

n. Exhibits strange or repetitive behaviour as chasing its own tail.

[never/nearly never/sometimes/often/always]

34. Indicate how often in below situations your dog shows one or more of these behaviours: seeks shelter behind your legs or an object, walks away, runs, freezes, whines, shakes or keeps its tail between its legs

a. When at the vet or groomer
b. When nearby other dogs
c. With children
d. With adults
e. When hearing loud noises
f. In traffic or at encountering strange objects
g. In new or unexpected situations

[never/nearly never/sometimes/often/always]

35. Indicate how often in below situations your dog shows one or more of these behaviours: barking, growling, raising lips, baring teeth, snapping, biting

a. When at the vet or groomer
b. When near its bowl, bone, toy
c. When nearby known dogs in its own home or garden
d. When nearby unknown dogs in its own home or garden
e. When nearby dogs outside its own home or garden
f. Directed at you or family member
g. Directed at children
h. Directed at adults

[never/nearly never /sometimes/often/always]

36. Have you visited a dog school with your dog?

[yes/no]

a. If so, how long?

[1-8 weeks/2-6 months/6-12 months/>1 year]

b. Which training aids were used?

[play/food/clicker/correction chain/other]

37. Does your dog learn what you wish him to, quickly?

[yes/no]

38. Are you able to influence behaviour of your dog with:

a. his normal food,

[yes/no]

b. tasty food,

[yes/no]

c. the promise of playing together or getting a ball?

[yes/no]

39. Is your dog quickly distracted by sounds, smells, things he sees?

[never/nearly never /sometimes/often/always]

40. Are you satisfied with the possibilities of walking the dog nearby your home?

[yes/no]

41. Is it possible to walk 30 minutes or more with the dog within ten minutes walking distance from you home?

[yes/no]

42. How much time do you spend walking on a weekday with your dog on average?

[<15 min/15-30 min/30-60 min/60-90 min/>90 min]

43. Are you satisfied with community policies on dogs?

[yes/no]

44. How much time is you dog at home alone per week or day on average?

[not at all/<8 hours per week/1-4 hours per day/4-6 hours per day/6-8 hours per day/8-10 hours per day/>10 hours per day]

45. How often do you play with your dog on average?

[not at all/<15 min per week/15-60 min per week/5-10 min per day/10-15 min per day/15-30 min per day/>30 min per day]

46. How often do you take care of your dog's coat?

[not at all/daily/weekly/monthly or less]

47. How often do you de-flea your dog?

[not at all/wearing flea collar/about once per quarter/about once per half year/about once per year]

48. How often do you deworm your dog?

[not at all/about once per quarter/about once per half year/about once per year]

49. How often do you give bones/chewing material to your dog?

[daily/nearly daily/few times per week/once per week/once per month/once per quarter/once per year/never]

50. Does your dog ever go out with a walking service?

[yes/no]

51. Do you clean after your dog in populated area?

[never/nearly never/sometimes/often/always]

52. Do you clean after your dog outside of populated area?

[never/nearly never/sometimes/often/always]

53. Do you put your dog on leash or in heel position when approaching a leashed dog?

[never/nearly never/sometimes/often/always]

54. Do you put your dog on leash or in heel position when approaching people in recreational

areas?

[never/nearly never/sometimes/often/always]
