# Supplementary material for: Dog ownership satisfaction determinants in the owner-dog relationship and the dog's behaviour
Source: PLoS One. 2018 Sep 20;13(9):e0204592. doi: 10.1371/journal.pone.0204592 (PMC6147508; doi:10.1371/journal.pone.0204592)
Supplement: S2 Appendix — Prevalence of dog aggression and two obedience behaviours based on the owner’s assessments of different situations (N = 968 to 972, with precise sample sizes between brackets). (PDF) [file pone.0204592.s002.pdf]

S2 Appendix - Prevalence of dog aggression and obedience in 968 to 972 Dutch dog owners

Prevalence of dog aggression and two obedience behaviours based on the owner's assessments of different situations (*N*=968 to 972, with precise sample sizes between brackets).

**Behaviour**	Sometimes, often, or always	Never or nearly never
Aggression at vet/groomer	8% (75)	92% (896)
Aggression near bowl, bone, toy	6% (57)	94% (915)
Aggression towards familiar dog on territory	17% (162)	83% (807)
Aggression towards unfamiliar dog on territory	36% (350)	64% (622)
Aggression towards dog off territory	32% (311)	68% (661)
Aggression towards owner/family member	2% (21)	98% (951)
Aggression towards child	6% (54)	94% (917)
Aggression towards adult	8% (79)	92% (889)
Obedience by coming when called	98% (948)	2% (24)
Disobedience by jumping up/pushing against people	38% (365)	62% (607)
